# No evidence of horizontal infection in horses kept in close contact with dogs experimentally infected with canine influenza A virus (H3N8)

**DOI:** 10.1186/1751-0147-54-25

**Published:** 2012-04-16

**Authors:** Takashi Yamanaka, Manabu Nemoto, Hiroshi Bannai, Koji Tsujimura, Takashi Kondo, Tomio Matsumura, Masanori Muranaka, Takanori Ueno, Yuta Kinoshita, Hidekazu Niwa, Kazuya IPJ Hidari, Takashi Suzuki

**Affiliations:** 1 Epizootic Research Center, Equine Research Institute, the Japan Racing Association, 1400-4 Shiba, Shimotsuke, Tochigi, 329-0412, Japan; 2 Department of Biochemistry, School of Pharmaceutical Sciences, Global COE Program, University of Shizuoka, 51-1 Yada, Suruga-ku, Shizuoka, 422-8526, Japan

**Keywords:** Canine influenza, Dog, H3N8, Horse, Interspecies transmission

## Abstract

**Background:**

Since equine influenza A virus (H3N8) was transmitted to dogs in the United States in 2004, the causative virus, which is called canine influenza A virus (CIV), has become widespread in dogs. To date, it has remained unclear whether or not CIV-infected dogs could transmit CIV to horses. To address this, we tested whether or not close contact between horses and dogs experimentally infected with CIV would result in its interspecies transmission.

**Methods:**

Three pairs of animals consisting of a dog inoculated with CIV (10^8.3^ egg infectious dose_50_/dog) and a healthy horse were kept together in individual stalls for 15 consecutive days. During the study, all the dogs and horses were clinically observed. Virus titres in nasal swab extracts and serological responses were also evaluated. In addition, all the animals were subjected to a gross pathological examination after euthanasia.

**Results:**

All three dogs inoculated with CIV exhibited clinical signs including, pyrexia, cough, nasal discharge, virus shedding and seroconversion. Gross pathology revealed lung consolidations in all the dogs, and *Streptococcus equi* subsp. *zooepidemicus* was isolated from the lesions. Meanwhile, none of the paired horses showed any clinical signs, virus shedding or seroconversion. Moreover, gross pathology revealed no lesions in the respiratory tracts including the lungs of the horses.

**Conclusions:**

These findings may indicate that a single dog infected with CIV is not sufficient to constitute a source of CIV infection in horses.

## 

Influenza A viruses have been isolated from a wide variety of animals, including humans, pigs, sea mammals, horses, poultry and aquatic birds. It is well known that aquatic birds serve as a natural reservoir from which all influenza A viruses have emerged [1]. It has generally been accepted that equine influenza A virus (EIV) subtype H3N8, which was first isolated from a diseased horse in Florida in the United States in 1963 [2], was also introduced into horses from aquatic birds [1]. EIV infection in a horse produces an acute respiratory disease [3]. EIV can have a profound economic impact on the horse industry causing a major disruption to horse racing and breeding because of its rapid spread. Thus, EIV still poses a significant threat to the equine industry [4].

Until recently dogs were not considered susceptible hosts of influenza A viruses [5]. However, there was an outbreak of respiratory disease among greyhounds in Florida in the United States at the beginning of 2004 [6]. A subsequent analysis of the causative virus revealed that the outbreak resulted from an interspecies transmission of contemporary EIV subtype H3N8 into dogs. After the outbreak in 2004, the causative virus, which came to be called canine influenza A virus (CIV), spread rapidly among dogs and probably became enzootic, at least in the United States [7,8].

Because CIV is genetically close to the ancestral form of EIV [6], it has been intriguing to learn whether CIV-infected dogs could be the source of the transmission of the H3N8 influenza A virus to the horse population. We previously demonstrated that the CIV A/canine/Colorado/30604/2006 (CO06) strain reduced proliferation ability and pathogenicity in horses compared with EIV, although CO06 (10^8.3^ egg infectious dose_50_/dog) is still infectious to horses via experimental inoculation using an ultrasonic nebulizer connected to a facemask [9]. All three horses exhibited seroconversion to CO06. Moreover, one of the three experimental horses shed live virus from the nostrils. Quintana et al. [10] also reported that the CIV-specific gene was detected in nasal swabs collected from one pony experimentally inoculated with CIV via aerosol inhalation. However, the experimental inoculation with the ultrasonic nebulizer seems unlikely to parallel natural infection in the field. Therefore, it remains unclear whether or not CIV-infected dogs could be the source of the H3N8 influenza A virus infection found in horses in the field. To address this, we undertook research to determine whether close contact between a CIV-infected dog and a horse could cause the interspecies transmission of CIV.

## Materials and methods

Three dogs (beagles, 11 months old) and three Thoroughbred horses (18–19 months old) were studied. All the animals were healthy and showed no serological evidence of prior H3N8 virus infection or vaccination in haemagglutination inhibition (HI) tests (HI titres <10) for antibodies to CO06 and EIV (A/equine/Ibaraki/1/2007, H3N8) (See below). CO06 was isolated from a diseased dog showing an acute respiratory sign in the United States. Each dog was randomly paired with a horse giving a total of three pairs. The dogs were inoculated with CO06 [10^8.3^ 50% egg infective doses (EID_50_)] by inhalation using an ultrasonic nebulizer (Soniclizer305, ATOM, Tokyo, Japan) connected to a facemask on Day 0 under sedation by intramuscular administration of medetomidine hydrochloride (10 μg/kg bodyweight; Domitor, Zenoaq, Fukushima, Japan).

Each pair remained in the same stall (3 m wide, 6.05 m deep and 4.1 m high) continuously from Day 0 to Day 14 as previously reported [11]. On Day 14, the dogs were euthanized. From Days 15 to 21, each horse was kept alone in the same stall. The rectal temperatures of all the animals were measured each morning during the experiment. A dog and a horse with rectal temperatures exceeding 39.5 and 38.8°C, respectively, were defined as having significant pyrexia in this study. We performed daily physical observations of the dogs and horses from Days −1 to 14 and from Days −1 to 21, respectively. The observation records for each animal were assigned scores as previously described by Jirjis et al. [12] and Toulemonde et al. [13] with slight modifications (Table 1). We calculated the total clinical scores for each animal each day.

**Table 1:** Clinical score assignment of respiratory disease

		Nasal discharge
0	No discharge	
0.5	Serous discharge	
1.0	Mucopurulent discharge	
Cough		
0	No cough	
0.5	Mild	
1.0	Moderate, persistent	
0	Absent	
2.0	Present	

Nasal samples were collected from the dogs using 3.0 × 6.0 mm absorbent cotton swabs (1P1501, JCB Industry, Tokyo, Japan) and from the horses using 1.0 × 1.5 cm absorbent cotton swabs (JMS menbou, Japan Medical Supply, Hiroshima, Japan) on a daily basis (from Days -1 to 14 and Days -1 to 21, respectively) and their extracts were titrated in 10-day-old embryonated hen’s eggs as previously described [11]. Briefly, the swabs collected from the horses and the dogs were immersed in 2.5 and 1.0 ml of transport medium [phosphate buffered saline (PBS, pH 7.2) supplemented with 0.6% tryptose phosphate broth, 500 unit/ml penicillin, 500 μg/ml streptomycin and 1.25 μg/ml amphotericin B), respectively. The swab samples in the transport medium were vortexed and briefly centrifuged to precipitate debris. Then, 200 μl of the supernatants that had been diluted at 1:10 (v/v) in transport medium were injected into the allantoic cavities of embryonated hen’s eggs (four eggs per sample). The allantoic fluid was harvested after 3 days of incubation at 34.0°C and examined for the presence of influenza A virus in a hemagglutination test using 0.5% hen’s red blood cells. The virus titres (log_10_EID_50_ /200 μl) were determined for nasal swab samples that were haemagglutination-positive [14].

The HI titres to CO06 of the sera collected from each dog or horse on Days −1, 9 and 14 or Days −1, 9, 14 and 21, respectively, were also measured as previously described [15]. Briefly, the antisera were treated with trypsin, heat and potassium metaperiodate to remove non-specific inhibitors. Then the required final dilution of the treated antiserum (1:10) was prepared and adsorbed with packed chicken erythrocytes. Two-fold dilutions of the antiserum with PBS were prepared; 25 μl of the diluted serum was used in each well of a microplate. 25 μl of virus containing 4 haemagglutination units was added to each well, and the microplate was incubated at room temperature for 30 min. Then 50 μl of 0.5% chicken erythrocytes was added to each well. The results were read after incubation at room temperature for 60 min. The HI antibody titres were determined by the reciprocal of the highest serum dilution that exhibited no haemagglutination.

The dogs and horses were examined by gross pathology on Days 14 and 21, respectively, after the euthanasia. If lesions were observed by gross pathology, they were subjected to further bacterial examinations. The samples were aseptically trimmed and then homogenized with nine times their volume of sterile distilled water. Subsequently, 100 μl of each sample was inoculated onto a blood agar plate and a MacConkey agar plate, and then incubated aerobically for 24 h at 37°C or anaerobically for 48 h also at 37°C. Bacterial identification was performed by employing Gram staining, morphological features, the catalase test, the oxidase test and commercial identification test kits (API, SYSMEX).

All experimental procedures were conducted in a biosafety level-3 facility and approved by the Animal Care Committee of the Equine Research Institute of the Japan Racing Association.

## Results and discussion

**Figure 1:**
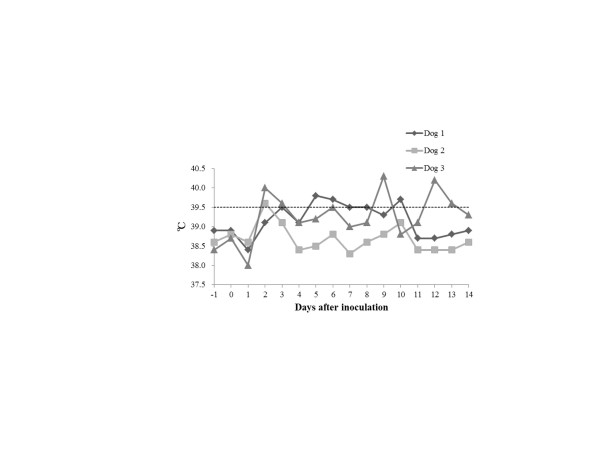
**Body temperatures of each dog.** The horizontal dotted line represents 39.5°C

**Figure 2:**
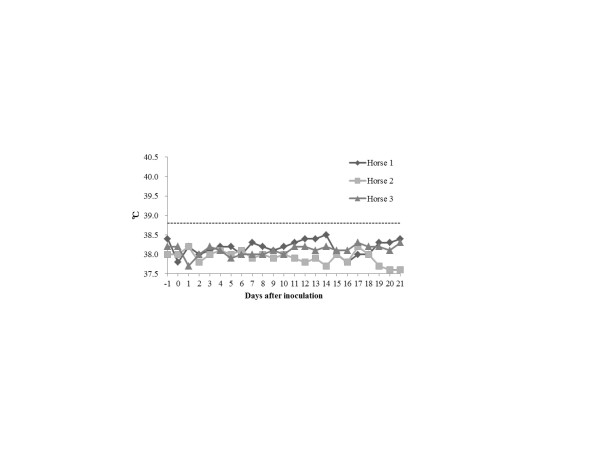
**Lung lesions in each of the infected dogs that were euthanized on Day 14**. Arrows point to the areas of lung consolidation

The rectal temperatures of the dogs are shown in Figure 1. All the dogs began showing significant pyrexia (≥39.5°C) on Day 2 or 3. Although Dog 2 exhibited significant pyrexia only for Day 2, Dogs 1 and 3 were pyrexial for six days during this study. The clinical scores of the dogs are listed in Table 2. All the dogs showed a range of clinical signs, including a nasal discharge and cough after inoculation. Although only Dog 2 showed a serous nasal discharge, Dogs 1 and 3 showed a mucopurulent nasal discharge for two days and seven days respectively after inoculation. Moreover, Dog 3 was observed sneezing on Day 10 and exhibited depression on Day 12. The results of virus titres of the nasal swab extracts collected from the dogs are presented in Table 3. The viruses were isolated from the nasal swabs collected from all the dogs for two to four days after inoculation. All the dogs showed seroconversion to CO06 in an HI test (Table 4). The gross pathology revealed lung consolidations that ranged from dark red to brown (Figure 2) in all the experimental dogs. *Streptococcus equi* subsp. *zooepidemicus* was isolated from the lung consolidations of Dogs 1, 2 and 3 (2.4 × 10^4^, 1.2 × 10^4^ and 7.4 × 10^5^ colony forming unit/g, respectively). It has been reported that secondary pneumonia induced by *Streptococcus equi* subsp. *zooepidemicus* was observed in diseased dogs during a CIV outbreak in Iowa in the United States in 2005 [16]. Collectively, these findings demonstrate that we reproduced the typical clinical features of field canine influenza experimentally infected with CIV.

**Table 2:** Clinical scores for each animal^a^

Days after inoculation	Dog 1	Horse 1	Dog 2	Horse 2	Dog 3	Horse 3
−1	0	0	0	0	0	0
0	0	0	0	0	0	0
1	0	0	0	0	0.5	0
2	0	0	1.5	0	2.0	0
3	1.0	0	0.5	0	0.5	0
4	0.5	0	0.5	0	0.5	0
5	0.5	0	0	0	0.5	0
6	1.0	0	0.5	0	1.0	0
7	0	0	0.5	0	0.5	0
8	1.0	0	0.5	0	2.5	0
9	1.0	0	0.5	0	2.0	0
10	2.0	0	1.0	0	3.5	0
11	0.5	0	0.5	0	0.5	0
12	0.5	0	0	0	2.0	0
13	1.5	0	0.5	0	1.5	0
14^b^	0	0	0	0	1.5	0
15	Nil	0	Nil	0	Nil	0
16	Nil	0	Nil	0	Nil	0
17	Nil	0	Nil	0	Nil	0
18	Nil	0	Nil	0	Nil	0
19	Nil	0	Nil	0	Nil	0
20	Nil	0	Nil	0	Nil	0
21^b^	Nil	0	Nil	0	Nil	0

^a^ Animals with the same number were paired.

^b^The dogs and horses were euthanized on Day 14 and 21, respectively, after sample collection

**Table 3:** Virus detection by egg culture and titre (log_10_EID_50_/200 μl) of nasal swab specimen collected daily from each animal

Days after inoculation	Dog 1	Horse 1	Dog 2	Horse 2	Dog 3	Horse 3
−1	-^a^	-	-	-	-	-
0	-	-	-	-	-	-
1	-	-	≤1.3	-	-	-
2	2.0	-	1.5	-	-	-
3	≤1.0	-	2.2	-	≤1.0	-
4	-	-	-	-	≤1.0	-
5	-	-	2.8	-	1.7	-
6	-	-	-	-	-	-
7	-	-	-	-	-	-
8	-	-	-	-	-	-
9	-	-	-	-	-	-
10	-	-	-	-	-	-
11	-	-	-	-	-	-
12	-	-	-	-	-	-
13	-	-	-	-	-	-
14^a^	-	-	-	-	-	-
15	Nil	-	Nil	-	Nil	-
16	Nil	-	Nil	-	Nil	-
17	Nil	-	Nil	-	Nil	-
18	Nil	-	Nil	-	Nil	-
19	Nil	-	Nil	-	Nil	-
20	Nil	-	Nil	-	Nil	-
21^c^	Nil	-	Nil	-	Nil	-

^a^ <0.7 (No CIV haemagglutination activity was detected from four eggs inoculated with 1:10 dilution of nasal swab specimen).

**Table 4:** HI titres of each animal

Days after inoculation	Dog 1	Horse 1	Dog 2	Horse 2	Dog 3	Horse 3
−1	<10	<10	<10	<10	<10	<10
9	40	<10	40	<10	20	<10
14	160	<10	640	<10	320	<10
21	Nil	<10	Nil	<10	Nil	<10

**Figure 3:**
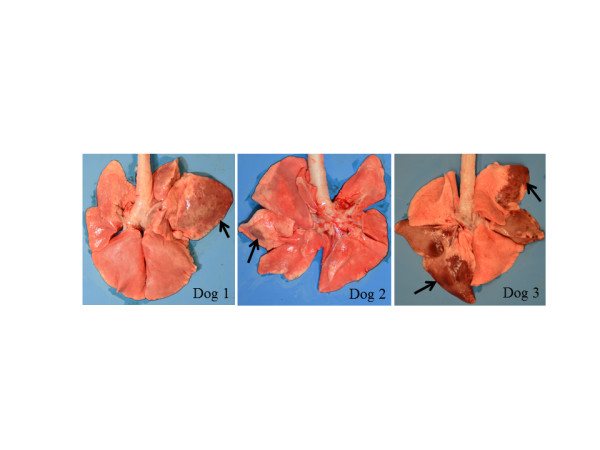
**Body temperatures of each horse**. The horizontal dotted line represents 38.8°C

Meanwhile, none of the paired horses showed any pyrexia (Figure 3) or other clinical signs (Table 2). No horses presented with virus shedding (Table 3) or seroconversion (Table 4) in this study. No lesions were observed in the respiratory tract including the lungs of the horses by gross pathology. Moreover, no specific gene of H3 subtype was detected in nasal swab specimens daily collected from each horse throughout this study by reverse transcription loop-mediated isothermal amplification assay (See Additional file 1) [17]. Thus, we conclude that there was no evidence suggesting the infection of horses with CIV in this study.

We have previously reported the feasibility of the close contact transmission of EIV (A/equine/Ibaraki/1/2007, H3N8) from a diseased horse to a paired dog [11]. The current result is opposite to that in the previous study. One reason for this may be the difference between the body sizes of dogs and horses. In terms of average body weight, the dogs at 12.7 kg were more than 25 times lighter than the horses (342 kg) at the beginning of this study. In fact, the highest titre of each dog during this study (range 10^1.7^ to 10^2.8^ EID_50_/200 μl, Table 3) was apparently lower than those of horses inoculated with EIV in the previous study (range 10^3.5^ to 10^4.3^ EID_50_/200 μl) [11], although the sampling conditions (swab size and medium volume) were admittedly different between dogs and horses. In turn, this could result in a difference in the total quantities of viral excretions into the air from the dogs and horses. The other reason could be the difference between the viral features of CIV and EIV. It has previously been reported that CO06 had reduced infectivity and pathogenicity in horses compared with A/equine/Ibaraki/1/2007 probably because of the reduction in the ability of CO06 to bind to *N*-glycolylneuraminic acid α2-3 galactose [9], which is predominantly expressed in the horse respiratory tract [18]. This may also contribute to the difference between the results of the previous study [11] and the current study. It has been reported that surveillance from 2005 to 2008 has provided no evidence of CIV infection among horses in the United States [19]. Our findings in this study are consistent with the phenomenon observed in the field.

## Conclusions

We demonstrated experimentally that close contact between a horse and a single dog infected with CIV did not lead to the interspecies transmission of CIV. This may indicate that a single dog infected with CIV is not sufficient to constitute a source of CIV infection in horses.

## Competing interests

The authors declare that they have no competing interests.

## Authors’ contributions

TY, MN, HB, KT and MM inoculated the dogs with CIV, performed the clinical observations and collected the samples. TY drafted the manuscript, processed the samples and measured the virus titres and HI titres. MM and TU carried out the gross pathology. YK and HN carried out the bacterial isolation and identification. TK, TM, KIPJH and TS participated in the design of the experiment and discussed the draft of the manuscript. We all read and approved the final manuscript.

## Supplementary Material

Additional file 1 Detection of viral specific gene by reverse transcription loop-mediated isothermal amplification (RT-LAMP) assay of nasal swab specimen collected daily from each horse. Click here for file

## References

[B1] Webster RG Bean WJ Gorman OT Chambers TM Kawaoka Y Evolution and ecology of influenza A viruses Microbiol Rev 1992 56 152 179 1579108 10.1128/mr.56.1.152-179.1992 PMC372859

[B2] Waddell GH Teigland MB Sigel MM A New Influenza Virus Associated with Equine Respiratory Disease J Am Vet Med Assoc 1963 143 587 590 14077956

[B3] van Maanen C Cullinane A Equine influenza virus infections: an update Vet Q 2002 24 79 94 10.1080/01652176.2002.9695127 12095083

[B4] Elton D Bryant N Facing the threat of equine influenza Equine Vet J 2011 43 250 258 10.1111/j.2042-3306.2010.00357.x 21492200

[B5] Buonavoglia C Martella V Canine respiratory viruses Vet Res 2007 38 355 373 10.1051/vetres:2006058 17296161

[B6] Crawford PC Dubovi EJ Castleman WL Stephenson I Gibbs EP Chen L Smith C Hill RC Ferro P Pompey J Transmission of equine influenza virus to dogs Science 2005 310 482 485 10.1126/science.1117950 16186182

[B7] Dubovi EJ Canine influenza Vet Clin North Am Small Anim Pract 2010 40 1063 1071 10.1016/j.cvsm.2010.07.005 20933136 PMC7132494

[B8] Dubovi EJ Njaa BL Canine influenza Vet Clin North Am Small Anim Pract 2008 38 827 835 10.1016/j.cvsm.2008.03.004 18501281

[B9] Yamanaka T Tsujimura K Kondo T Matsumura T Ishida H Kiso M Hidari KI Suzuki T Infectivity and pathogenicity of canine H3N8 influenza A virus in horses Influenza Other Respi Viruses 2010 4 345 351 10.1111/j.1750-2659.2010.00157.x 20958928 PMC4634615

[B10] Quintana AM Hussey SB Burr EC Pecoraro HL Annis KM Rao S Landolt GA Evaluation of infectivity of a canine lineage H3N8 influenza A virus in ponies and in primary equine respiratory epithelial cells Am J Vet Res 2011 72 1071 1078 10.2460/ajvr.72.8.1071 21801065

[B11] Yamanaka T Nemoto M Tsujimura K Kondo T Matsumura T Interspecies transmission of equine influenza virus (H3N8) to dogs by close contact with experimentally infected horses Vet Microbiol 2009 139 351 355 10.1016/j.vetmic.2009.06.015 19596528

[B12] Jirjis FF Deshpande MS Tubbs AL Jayappa H Lakshmanan N Wasmoen TL Transmission of canine influenza virus (H3N8) among susceptible dogs Vet Microbiol 2010 144 303 309 10.1016/j.vetmic.2010.02.029 20347235

[B13] Edlund Toulemonde C Daly J Sindle T Guigal PM Audonnet JC Minke JM Efficacy of a recombinant equine influenza vaccine against challenge with an American lineage H3N8 influenza virus responsible for the 2003 outbreak in the United Kingdom Vet Rec 2005 156 367 371 15816180 10.1136/vr.156.12.367

[B14] Reed LJ Müench H A simple method of estimating fifty per cent of endpoint AM J Hyg 1938 27 493

[B15] Yamanaka T Bannnai H Nemoto M Tsujimura K Kondo T Matsumura T Antibody Responses Induced by Japanese Whole Inactivated Vaccines against Equine Influenza Virus (H3N8) Belonging to Florida Sublineage Clade2 J Vet Med Sci 2011 73 483 485 10.1292/jvms.10-0408 21099188

[B16] Yoon KJ Cooper VL Schwartz KJ Harmon KM Kim WI Janke BH Strohbehn J Butts D Troutman J Influenza virus infection in racing greyhounds Emerg Infect Dis 2005 11 1974 1976 16485496 10.3201/eid1112.050810 PMC3367648

[B17] Nemoto M Yamanaka T Bannai H Tsujimura K Kondo T Matsumura T Development and evaluation of a reverse transcription loop-mediated isothermal amplification assay for H3N8 equine influenza virus J Virol Methods 2011 178 239 242 10.1016/j.jviromet.2011.07.015 21907240

[B18] Suzuki Y Ito T Suzuki T Holland RE Chambers TM Kiso M Ishida H Kawaoka Y Sialic acid species as a determinant of the host range of influenza A viruses J Virol 2000 74 11825 11831 10.1128/JVI.74.24.11825-11831.2000 11090182 PMC112465

[B19] Rivailler P Perry IA Jang Y Davis CT Chen LM Dubovi EJ Donis RO Evolution of canine and equine influenza (H3N8) viruses co-circulating between 2005 and 2008 Virology 2010 408 71 79 10.1016/j.virol.2010.08.022 20880564

