# Supplementary material for: No evidence of horizontal infection in horses kept in close contact with dogs experimentally infected with canine influenza A virus (H3N8)
Source: Acta Vet Scand. 2012 Apr 16;54(1):25. doi: 10.1186/1751-0147-54-25 (PMC3416777; doi:10.1186/1751-0147-54-25)
Supplement: Additional file 1 — Detection of viral specific gene by reverse transcription loop-mediated isothermal amplification (RT-LAMP) assay of nasal swab specimen collected daily from each horse. [file 1751-0147-54-25-S1.docx]

Additional file 1:

**Detection of viral specific gene by reverse transcription loop-mediated isothermal amplification (RT-LAMP) assay of nasal swab specimen collected daily from each horse.**

| Days after inoculation | Horse 1 | Horse 2 | Horse 3 |
| --- | --- | --- | --- |
| -1 | -^a^ | - | - |
| 0 | - | - | - |
| 1 | - | - | - |
| 2 | - | - | - |
| 3 | - | - | - |
| 4 | - | - | - |
| 5 | - | - | - |
| 6 | - | - | - |
| 7 | - | - | - |
| 8 | - | - | - |
| 9 | - | - | - |
| 10 | - | - | - |
| 11 | - | - | - |
| 12 | - | - | - |
| 13 | - | - | - |
| 14^a^ | - | - | - |
| 15 | - | - | - |
| 16 | - | - | - |
| 17 | - | - | - |
| 18 | - | - | - |
| 19 | - | - | - |
| 20 | - | - | - |
| 21 | - | - | - |

^a^ negative.

RNA extraction and RT-LAMP assay were conducted according to our previous study [17]. The assay was validated using CO06 as a positive control.
